# Robotic versus Laparoscopic Approach in Colonic Resections for Cancer and Benign Diseases: Systematic Review and Meta-Analysis

**DOI:** 10.1371/journal.pone.0134062

**Published:** 2015-07-27

**Authors:** Stefano Trastulli, Roberto Cirocchi, Jacopo Desiderio, Andrea Coratti, Salvatore Guarino, Claudio Renzi, Alessia Corsi, Carlo Boselli, Alberto Santoro, Liliana Minelli, Amilcare Parisi

**Affiliations:** 1 Department of Gastrointestinal Surgery and Liver Unit, St. Maria Hospital, Terni, Italy; 2 Department of Oncology, Division of Oncological and Robotic Surgery, Careggi University Hospital, Florence, Italy; 3 Department of Surgical Science, “Sapienza” University, Rome, Italy; 4 Department of General and Oncologic Surgery, University of Perugia, Perugia, Italy; 5 Department of Experimental Medicine, Public Health Section, University of Perugia. Perugia, Italy; Heinrich-Heine-University and University Hospital Duesseldorf, GERMANY

## Abstract

**Objectives:**

The aim of this systematic review and meta-analysis is to compare robotic colectomy (RC) with laparoscopic colectomy (LC) in terms of intraoperative and postoperative outcomes.

**Materials and Methods:**

A systematic literature search was performed to retrieve comparative studies of robotic and laparoscopic colectomy. The databases searched were PubMed, Embase and the Cochrane Central Register of Controlled Trials from January 2000 to October 2014. The Odds ratio, Risk difference and Mean difference were used as the summary statistics.

**Results:**

A total of 12 studies, which included a total of 4,148 patients who had undergone robotic or laparoscopic colectomy, were included and analyzed. RC demonstrated a longer operative time (MD 41.52, P<0.00001) and higher cost (MD 2.42, P<0.00001) than did LC. The time to first flatus passage (MD -0.51, P = 0.003) and the length of hospital stay (MD -0.68, P = 0.01) were significantly shorter after RC. Additionally, the intraoperative blood loss (MD -16.82, P<0.00001) was significantly less in RC. There was also a significantly lower incidence of overall postoperative complications (OR 0.74, P = 0.02) and wound infections (RD -0.02, P = 0.03) after RC. No differences in the postoperative ileus, in the anastomotic leak, or in the conversion to open surgery rate and in the number of harvested lymph nodes outcomes were found between the approaches.

**Conclusions:**

The present meta-analysis, mainly based on observational studies, suggests that RC is more time-consuming and expensive than laparoscopy but that it results in faster recovery of bowel function, a shorter hospital stay, less blood loss and lower rates of both overall postoperative complications and wound infections.

## Introduction

Laparoscopy has definitively emerged as the gold standard approach for the treatment of both malignant [1, 2] diseases of the colon and benign diseases of the colon, such as in the elective surgical treatment of diverticular disease [3].

Despite swift technological advances and widespread use, laparoscopy has some limitations, which are mainly related to the 2-dimensional view of the operating field, physiological tremor of the camera operator and lack of ergonomic design of the instruments (which increases operator and assistant fatigue).

The purpose of introducing robotic technology into surgical practice was to overcome the technical disadvantages of laparoscopy.

Robot use in colorectal surgery has been investigated more thoroughly for rectal cancer surgery than for colonic surgery [4–7], but some authors [8–11] have suggested that robotic surgery could provide advantages when performing some colonic resection steps, such as splenic flexure takedown and intra-corporeal suturing (intestinal anastomoses), which may also improve the accuracy of vascular pedicle dissection and lymphadenectomy.

To date, it is unclear whether these theoretical advantages translate into clinical benefits. The role of robot use in colonic surgery remains a matter of debate, particularly its cost-effectiveness [12].

A growing number of comparative studies have provided contradictory data. Some of these studies have found that robotic colonic surgery does not have any advantages compared to laparoscopy and that it is more time-consuming and cost expensive [13–15]. Other studies have shown that the robotic approach provides better recovery outcomes, lower postoperative complications and shorter operating times [16–18]. An earlier systematic review with meta-analysis on robotic colorectal surgery analyzed the outcomes of robotic versus laparoscopic colonic resections. This was only a subgroup analysis considering a total of 269 colectomies. It found no differences between the approaches, except for a longer operative time in the robotic group [19]. Petrucciani et al. recently conducted a meta-analysis comparing the robotic versus laparoscopic approach and focusing only on right colectomies. They included a total of six studies with a limited sample size (total of 168 patients in the robotic group and 348 in the laparoscopic group). The meta-analysis showed no differences between robotic and laparoscopic approach in the analyzed outcomes, except for a longer operative time for robotic right colectomy and the authors did not perform sensitivity or subgroup analysis [20]. Other well-conducted reviews, some of which were systematic but lacked a meta-analysis [12, 21], were limited because they were conducted during a period with a small number of published comparative studies on robotic versus laparoscopic colectomies.

Because of this background, our aim was to perform an up-to-date systematic review and meta-analysis of the literature to compare robotic versus laparoscopic colectomies (considering both right and left colectomies) performed on patients with malignant or benign diseases in terms of intraoperative and postoperative outcomes and costs.

## Materials and Methods

The present systematic review and meta-analysis was conducted following the instructions suggested in the Preferred Reporting Items for Systematic Reviews and Meta-Analyses (PRISMA) statement [22] and the Cochrane Handbook of Systematic Review [23].

### Search strategy

We conducted searches in the following electronic databases: PubMed, EMBASE and the Cochrane Central Register of Controlled Trials. We searched for studies comparing robotic and laparoscopic colectomies in patients affected by either malignant or benign diseases of the colon that were potentially eligible for the inclusion in this systematic review and were published from January 2000 to October 2014. The following search terms were used in various combinations: “Robot”, “Robot-assisted”, “Colon”, “Colorectal”, “Colonic”, “Right colectomy”, “Left colectomy”, “Sigmoidectomy”, “Transverse”, “Sigmoid” and “Hemicolectomy”. We used both free text and MeSH searches for keywords. The list of references in each eligible article was manually evaluated to determine studies of interest for this review.

The selected abstracts from the literature searches were independently evaluated by three authors, and the discrepancies, when present, were discussed and resolved with the consensus of the three authors. Only articles with both abstracts and full text in English were included.

The full text of the potentially eligible articles was obtained and then independently analyzed by the three authors to confirm its eligibility on the basis of the inclusion and exclusion criteria of this systematic review. Possible discrepancies were collegially discussed by the authors.

### Studies selection and inclusion and exclusion criteria

The inclusion criteria for this systematic review were randomized and non-randomized studies comparing patients undergoing resections of any portion of the colon (cecum, ascending, transverse, descending and sigma) independent from the extension of the resection with a robotic approach (full robotic or robotic assisted) versus a laparoscopic one and studies reporting data for at least one of the considered outcomes in patients affected by either malignant or benign diseases of the colon. Studies comparing the robotic and laparoscopic procedures that were performed with a single port or hand-assisted laparoscopic approach were also considered eligible.

The following exclusion criteria were considered: procedures of anterior resection of the rectum or abdomino-perineal resections performed for rectal cancer or benign rectal diseases, studies with fewer than 10 patients enrolled in each treatment group and studies reporting robotic procedures that were performed with a different robot other than Da Vinci (Intuitive Surgical, Mountain View, Sunnyvale, CA, USA).

In the cases in which authors and/or institutions overlapped between two or more studies, the corresponding authors were contacted; in the case of no response, only the most recent study was considered.

### Data Extraction

Once the full texts of the studies included in the systematic review were obtained, the data of interest were independently extracted and compared by the three authors using a predefined spreadsheet. In the case of discrepancies, the authors revised the extraction process and discussed the data before reaching a consensus.

The primary objective of this systematic review was to evaluate whether the robotic colon resection is capable of significantly reducing the length of hospital stay and the postoperative morbidity compared with the laparoscopic approach.

The primary outcomes were: length of hospital stay and overall postoperative complications.

The secondary outcomes considered were:

- Operative time
- Conversion to open surgery rate
- Intraoperative blood loss
- Time to first flatus
- Number of harvested lymph nodes
- Anastomotic leak rate
- Wound infections rate
- Postoperative ileus rate
- Costs

### Assessment of methodological quality and bias risk of the included studies

The methodological quality of the randomized studies was determined using the modified Jadad scale [24, 25], while for the observational studies the revised and modified grading system of the Scottish Intercollegiate Guidelines Network was used [26, 27]. For the included randomized studies, the Cochrane Collaboration’s tool for assessing the risk of bias was used [23].

### Statistical Analysis

The dichotomous outcomes were analyzed using the Odds ratio (OR) as the summary statistics with the Mantel-Haenszel method [28, 29]. The continuous outcomes were analyzed using the Mean difference (MD) with the generic inverse variance method. In the case of studies with dichotomous outcomes with 0 events in each of the treatment groups, the Risk Difference (RD) as a summary statistic was used to also include them in the estimated effect. Nevertheless, a sensitivity analysis using the OR was also performed in these cases.

Statistical heterogeneity was evaluated with a Chi-squared test [30] (statistical heterogeneity was defined as a P value <0.05) and a Higgins I^2^ [31] test. The Higgins I^2^ test measured inconsistency of the data. Values of < 25, between 25–50 and > 50% were defined as low, moderate or high, respectively. In cases of low or moderate inconsistency, the data were analyzed with the Fixed Effect model. In case of high heterogeneity, the data were analyzed with the Random Effect model [32].

In the studies in which continuous outcomes were reported as Medians and Ranges, the mean and standard deviation were calculated using a method suggested by Hozo et al [33].

The cost analysis was performed by discounting costs to those from 2013 with a 3% rate, as indicated in the panel on cost-effectiveness in health and medicine [34].

Moreover, for the studies reporting only the mean and not the range or standard deviation, we contacted the corresponding author of each study to obtain the necessary information. In the case of no response, these values were estimated using different methods, including the use of the T-values, P-values, confidence intervals, F-values, standard errors or imputation methods [23]. Publication bias was evaluated through the construction of funnel plots. All statistical analyses were performed using the Review Manager (RevMan) software, version 5.2 (Copenhagen: Nordic Cochrane Centre, Cochrane Collaboration, 2011).

### Subgroup analysis

In the present meta-analysis, in addition to the main analysis (performed comparing robotic and laparoscopic procedures for cancer and benign diseases from all of the included studies), the following subgroup analyses were performed:

- Procedures performed for cancer
- Right colectomy procedures
- Left colectomy procedures.

### Sensitivity analysis

We planned to perform a sensitivity analysis for each of the investigated outcomes excluding from the main analysis: 1) studies in which the data (mean and/or standard deviation) required for the meta-analysis were estimated, 2) studies performed based on a national database, 3) studies using single port procedures or hand-assisted procedures, 4) studies with low methodological quality (with a score <8 points on the modified grading system of the Scottish Intercollegiate Guidelines Network or a score <6 points on the modified Jadad Scale), 5) studies in which in at least one of the groups accounted for less than 20 patients and 6) randomized clinical trials.

## Results

**Fig 1:**
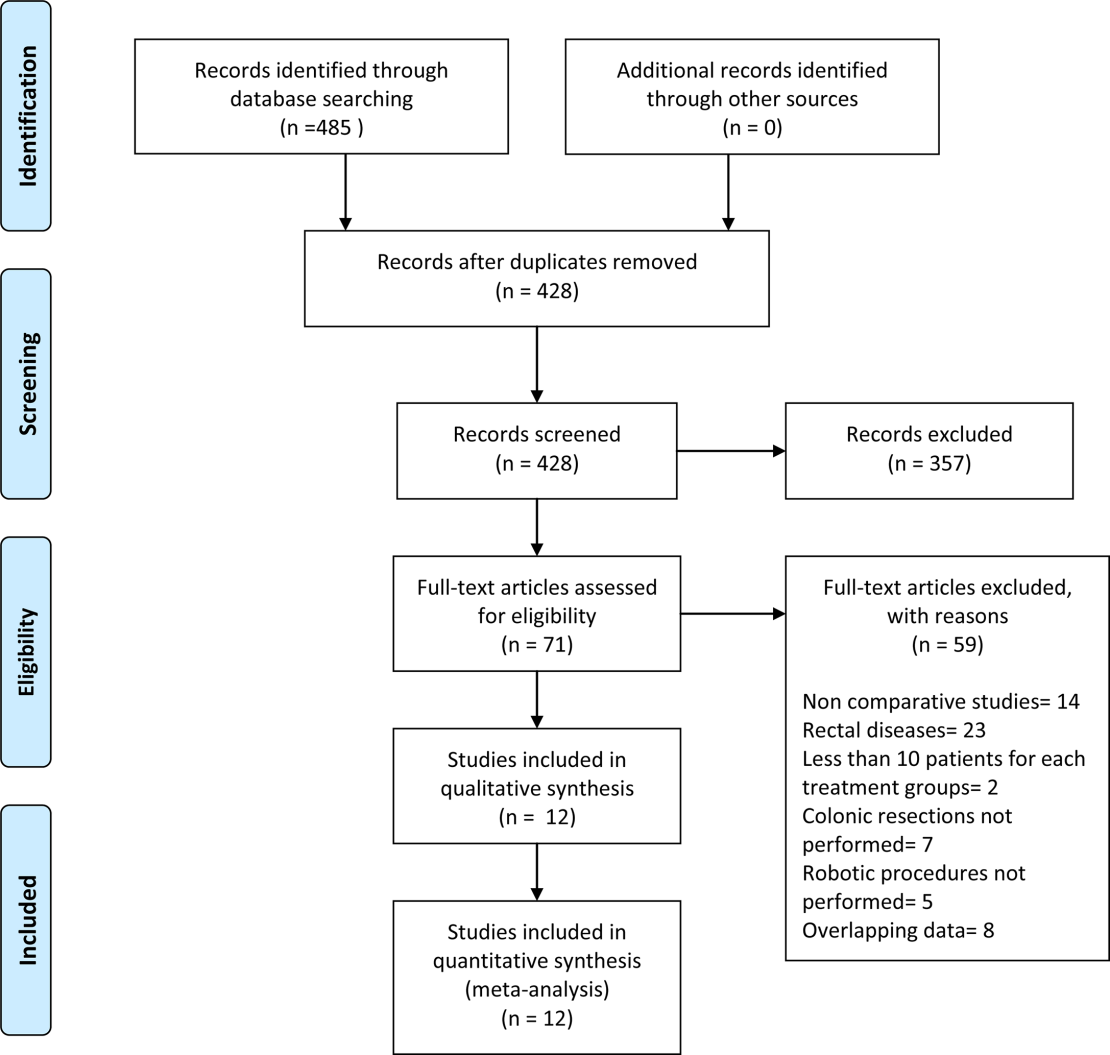
Flowchart for records selection process of the systematic review.

The bibliographic research identified a total of 485 records (Fig 1). Of these, 414 were excluded because they were duplicated or because they did not meet the inclusion criteria based on either the title or the content of the abstract. Seventy-one full-text articles were evaluated, and, of these, 59 were excluded because of overlap between the patients or because they were irrelevant based on the inclusion/exclusion criteria. Twelve studies [13–18, 35–40] met the inclusion criteria and were therefore included in the present systematic review and meta-analysis.

### Study characteristics

The eligible studies comprised a total of 4,148 patients, of which 744 (18%) underwent robotic colectomies and 3,404 (82%) underwent laparoscopic colectomies.

The characteristics of the included studies are summarized in Table 1. Among the included studies, only one was a randomized controlled trial [14], whereas the remaining studies were all retrospective observational studies, except the studies by Bertani et al [39] and Casillas et al. [17] which were a prospective non-randomized studies. The study by Casillas et al. [17] presented results that were adjusted by a propensity score calculation. We decided to include the large United States (US) national database study by Tyler et al. in this meta-analysis [15]. This database study was a retrospective analysis of the Nationwide Inpatient Sample (NIS) database. We did not include other studies based on the US national database [41, 42], not only because of the risk of overlapping institutions and patients but also because the study by Tyler et al. focused only on colectomies and included only patients who were treated in institutions that had performed at least 1 robotic colectomy over the study period [15]. The study by Tyler et al. presented results that were risk-adjusted on the basis of patients and hospitals factors.

**Table 1:** Characteristics of the included studies.

Author/Reference	Year	Country	Study design	Location	Study sample size, RC/LC	Gender (m, f), RC/LC	Age^†^, RC/LC	BMI^†^, RC/LC	ASA score 1-2-3-4, RC/LC	Cancer patients (%), RC/LC
Trastulli [16]	2015	Italy	R	Right colon	102/134	56, 46/77, 57	68.8/71	25.6/25.8	8-55-39-0/12-69-53-0	86.2% ^±^/89.5% ^±^
Rawlings^a^ [35]	2007	USA	R	Right colon	17/15	8, 9/6, 9	64.6/63.1	25.7/28.3	NR	11.8%/40%
Rawlings^b^ [35]	2007	USA	R	Left colon	13/12	6, 7/6, 6	61.2/60.3	27.8/27.8	NR	23%/16.7%
de Souza [13]	2012	USA	R	Right colon	40/135	22, 18/62, 73	71.35/65.32	27.33/26.57	5-14-20-1/17-67-48-3	45%^±^/48.9%^±^
Lujan [36]	2011	USA	R	Right colon	22/25	8, 14/10, 15	71.88/72.6	31.44/27.88	NR	45.4%/48%
Morpurgo [37]	2013	Italy	R	Right colon	48/48	27, 21/16, 32	68/74	25/28	8-28-12-0/4-26-17-1	100%/100%
Park [14]	2012	Korea	RCT	Right colon	35/35	14, 21/16, 19	62·8/66·5	24.4/23·8	15-16-4-0/21-12-2-0	100%/100%
Deutsch [38]	2012	USA	R	Right colon	18/47	12, 6/25, 22	65.2/70.8	25/28	14^#^/23^#^	27.8%/51%
Bertani [39]	2011	Italy	PNR	Right/Left colon	34/30	16, 18/17, 13	62.5/60	26.1/24.6	29^#^/27^#^	100%/100%
Helvind [18]	2013	Denmark	R	Right/Left colon	101/162	43, 58/69, 93	72.2/75.3	24.6/24.9	13-71-15-2/24-104-32-2	100%/100%
Casillas^a^ [17]	2014	USA	PNR*	Right colon	52/110	25, 27/69, 41	65/71	26.9/27	1-31-20-0/2-60-44-4	100%/100%
Casillas ^b^ [17]	2014	USA	PNR*	Left colon	68/82	38, 30/37, 45	56/60	28.3/28.4	0-56-12-0/2-62-18-0	31%/35.3%
Tyler [15]	2013	USA	R	Right/Left colon	160/2423	NR	NR	NR	NR	NR
Lim [40]	2013	Korea	R	Left colon	34/146	23, 11/87, 59	59.6/59.7	24.8/23.8	19-13-2-0/107-33-6-0	100%/100%

^a^ Right colectomy data sets

^b^ Left colectomy data sets

^†^ Mean or Median

^±^ Data on resections for cancer were separately reported in the manuscript

* Propensity score analysis

^#^ Total patients with ASA score 1 or 2

N: Number of patients; m: male; f: female; BMI: Body Mass Index; ASA: American Society of Anaesthesiology; R: Retrospective; RCT: Randomized controlled trial; PNR: Prospective non randomized; NR: Not Reported

The included studies were performed in the USA, Europe and Asia. Five of the studies enrolled only cancer patients [14, 17, 18, 37, 39, 40], whereas two of the studies reported data on oncological outcomes for patients with malignancy in a subgroup analysis [13, 16]. In the study by Casillas et al. [17] only the patients in the right colectomy group were all operated for cancer. All of the remaining studies included patients with colonic cancer, but the data were inseparable from the data derived from patients with benign disease [15, 35, 36, 38].

The technical characteristics of the surgical procedures performed in each of the included studies were reported in S1 Table. No studies clearly reported the use of the hand-assisted laparoscopic technique. One study [38] reported procedures performed using the single port technique in 3 of the 18 patients who received robotic procedures (17%) and in 2 of the 47 patients who received laparoscopic procedures (4.2%).

Only the studies by Rawlings et al.[35] and Deutsch et al.[38] had at least one treatment group with fewer than 20 patients.

The results of the meta-analysis for dichotomous and continuous investigated outcomes and the subgroup analyses are summarized in Tables 2 and 3, respectively. The results of the sensitivity analysis are summarized in Tables 4 and 5.

**Table 2:** Results of the meta-analysis for continuous outcomes.

Outcomes	Group or Subgroup	Set of data	N patients, RC/LC	Analysis model / Effect measure	I^2^	Summary Statistics	95% CI	P value
**Operative time**	**Total**	13	584/981	RE/MD	93%	41.52	23.59 to 59.45	***<0*.*00001***
	**Right colon**	8	334/549	RE/MD	90%	52.32	34.21 to 70.43	***<0*.*00001***
	**Left colon**	3	115/240	RE/MD	85%	49.01	10.53 to 87.49	***0*.*01***
	**Cancer**	6	304/531	RE/MD	94%	30.47	0.71 to 60.24	***0*.*04***
**Estimated blood loss**	**Total**	11	435/771	FE/ MD	27%	-16.82	-23.00 to -10.64	***<0*.*00001***
	**Right colon**	7	286/501	FE/ MD	9%	-18.28	-26.84 to -9.73	***<0*.*0001***
	**Left colon**	3	115/240	FE/ MD	43%	-16.17	-25.16 to -7.17	***0*.*0004***
	**Cancer**	4	155/321	FE/ MD	33%	-17.74	-25.29 to -10.18	***<0*.*00001***
**Time to first flatus**	**Total**	5	253/393	RE/ MD	66%	-0.51	-0.84 to -0.18	***0*.*003***
	**Right colon**	3	185/217	FE/MD	23%	-0.76	-0.99 to -0.54	***<0*.*00001***
	**Left colon**	1	34/146	FE/MD	-	-0.31	-0.64 to 0.02	0.06
	**Cancer**	4	151/259	RE/ MD	71%	-0.43	-0.89 to 0.02	0.06
**Length of hospital stay**	**Total**	14	675/3263	RE/MD	64%	-0.68	-1.20 to -0.16	***0*.*01***
	**Right colon**	8	301/468	RE/ MD	71%	-0.74	-1.61to 0.13	0.10
	**Left colon**	3	79/180	FE/ MD	47%	-0.85	-1.40 to -0.29	***0*.*003***
	**Cancer**	6	271/450	FE/ MD	8%	-0.65	-1.09 to -0.22	***0*.*003***
**Number of harvested lymph nodes**	**Total**	10	434/737	RE/ MD	60%	-0.83	-2.68 to 1.03	0.38
	**Right colon**	6	251/373	FE/MD	5%	-1.58	-3.09 to -0.07	***0*.*04***
	**Left colon**	2	48/172	RE/MD	90%	0.75	-6.60 to 8.10	0.84
	**Cancer**	8	384/673	RE/ MD	59%	-0.22	-2.27 to 1.83	0.84
**Costs**	**Total**	5	255/2576	FE/ MD	20%	2.42	1.74 to 3.11	***<0*.*00001***
	**Right colon**	3	82/141	FE/MD	0%	2.02	1.24 to 2.80	***<0*.*00001***
	**Left colon**	1	12/13	FE/MD	-	2.03	-7.33 to 11.39	0.67
	**Cancer**	1	35/35	FE/ MD	-	2.03	1.20 to 2.86	***<0*.*00001***

FE: Fixed Effect; RE: Random Effect; MD: Mean Difference; CI: Confidence interval

**Table 3:** Results of the meta-analysis for dichotomous outcomes.

Outcomes	Group or Subgroup	Set of data	N patients, RC/LC	Analysis model / Effect measure	I^2^	Summary Statistics	95% CI	P value
**Conversion to open**	**Total**	13	584/981	FE/ OR	20%	0.67	0.39 to 1.15	0.15
	Total *	13	584/981	FE/ RD	21%	-0.02	-0.04 to 0.00	0.13
	**Right colon**	8	334/549	FE/ OR	0%	0.38	0.17 to 0.87	***0*.*02***
	Right colon *	8	334/549	FE/ RD	45%	-0.03	-0.06 to -0.01	***0*.*02***
	**Left colon**	3	115/240	FE/ OR	1%	0.87	0.29 to 2.57	0.80
	**Cancer**	6	304/531	FE/ OR	25%	0.91	0.40 to 2.05	0.81
	Cancer *	6	304/531	FE/ RD	1%	-0.00	-0.03 to 0.02	0.72
**Overall postoperative complications**	**Total**	13	584/982	FE/OR	0%	0.74	0.57 to 0.95	***0*.*02***
	**Right colon**	8	334/550	FE/ OR	4%	0.70	0.50 to 0.96	***0*.*03***
	**Left colon**	3	115/240	FE/ OR	0%	0.64	0.32 to 1.29	0.21
	**Cancer**	6	304/531	FE/ OR	15%	0.62	0.43 to 0.90	***0*.*01***
**Anastomotic leak**	**Total**	13	584/981	FE/ OR	0%	0.70	0.37 to 1.30	0.26
	Total*	13	584/981	FE/ RD	0%	-0.01	-0.03 to 0.01	0.20
	**Right colon**	8	334/549	FE/ OR	16%	0.76	0.31 to 1.86	0.55
	Right colon*	8	334/549	FE/ RD	28%	-0.01	-0.03 to 0.02	0.51
	**Left colon**	3	115/240	FE/ OR	0%	0.31	0.05 to 1.84	0.20
	**Cancer**	6	304/531	FE/ OR	0%	0.58	0.26 to 1.29	0.18
**Postoperative ileus**	**Total**	12	609/3212	FE/ OR	0%	0.71	0.48 to 1.05	0.08
	Total *	12	609/3212	FE/ RD	3%	-0.02	-0.05 to 0.00	0.06
	**Right colon**	8	334/549	FE/ OR	0%	0.54	0.28 to 1.06	0.07
	Right colon *	8	334/549	FE/ RD	18%	-0.03	-0.06 to 0.00	0.05
	**Left colon**	3	115/240	RE/ OR	77%	1.56	0.05 to 47.03	0.80
	Left colon *	3	115/240	FE/ RD	37%	0.00	-0.04 to 0.05	0.92
	**Cancer**	4	169/339	RE/ OR	71%	1.04	0.08 to 13.66	0.98
	Cancer *	4	169/339	RE/ RD	67%	-0.01	-0.07 to 0.04	0.67
**Wound Infection**	**Total**	13	584/981	FE/ OR	0%	0.59	0.35 to 0.99	***0*.*04***
	Total *	13	584/981	FE/ RD	0%	-0.02	-0.05 to -0.00	***0*.*03***
	**Right colon**	8	334/549	FE/ OR	0%	0.67	0.36 to 1.26	0.21
	Right colon*	8	334/549	FE/ RD	0%	-0.02	-0.05 to 0.01	0.21
	**Left colon**	3	115/240	FE/ OR	0%	0.50	0.15 to1.62	0.25
	**Cancer**	6	304/531	FE/ OR	0%	0.54	0.26 to 1.14	0.11
	Cancer*	6	304/531	FE/ RD	0%	-0.03	-0.06 to 0.00	0.07

FE: Fixed Effect; RE: Random Effect; OR: Odds Ratio; RD: Risk Difference; CI: Confidence interval

*: Analysis performed with the Risk Difference (RD) as a summary statistic to also include in the estimated effect the studies with dichotomous outcomes with 0 events in each of the treatment groups.

**Table 4:** Results of the sensitivity analysis for continuous outcomes.

Outcomes	Group or Subgroup	Set of data	N patients, RC/LC	Analysis model / Effect measure	I^2^	Summary Statistics	95% CI	P value
**Operative time**	**Sensitivity analysis** ^**1**^	9	329/597	RE/MD	87%	46.65	27.36 to 65.94	***<0*.*00001***
	**Sensitivity analysis** ^**3**^	10	536/907	RE/MD	93%	47.01	28.08 to 65.95	***<0*.*00001***
	**Sensitivity analysis** ^**4**^	12	549/946	RE/MD	93%	39.50	20.29 to 58.71	***<0*.*0001***
**Estimated blood loss**	**Sensitivity analysis** ^**1**^	7	241/414	FE/ MD	15%	-18.99	-25.51 to -12.46	***<0*.*00001***
	**Sensitivity analysis** ^**3**^	8	387/697	FE/ MD	0%	-16.15	-22.66 to -9.63	***<0*.*00001***
	**Sensitivity analysis** ^**4**^	10	400/736	FE/MD	32%	-15.72	-22.67 to -8.78	***<0*.*00001***
**Time to first flatus**	**Sensitivity analysis** ^**1**^	4	219/363	RE/MD	60%	-0.62	-0.94 to -0.29	***0*.*0002***
	**Sensitivity analysis** ^**4**^	4	218/358	RE/MD	74%	-0.53	-0.90 to -0.16	***0*.*005***
**Length of hospital stay**	**Sensitivity analysis** ^**1**^	8	289/462	FE/ MD	49%	-1.12	-1.51 to -0.74	***<0*.*00001***
	**Sensitivity analysis** ^**2**^	13	515/840	RE/ MD	59%	-0.77	-1.36 to -0.19	***0*.*010***
	**Sensitivity analysis** ^**3**^	10	608/3160	RE/ MD	73%	-0.71	-1.28 to -0.13	***0*.*02***
	**Sensitivity analysis** ^**4**^	12	640/3228	RE/MD	67%	-0.70	-1.24 to -0.15	***0*.*01***
**Number of harvested lymph nodes**	**Sensitivity analysis** ^**1**^	5	241/388	RE/MD	63%	0.14	-2.75 to 3.03	0.92
	**Sensitivity analysis** ^**3**^	8	402/645	RE/ MD	60%	-0.21	-2.23 to 1.82	0.84
	**Sensitivity analysis** ^**4**^	9	399/702	RE/MD	65%	-0.95	-2.92 to 1.02	0.34
**Costs**	**Sensitivity analysis** ^**1**^	3	65/62	FE/MD	0%	1.99	1.20 to 2.77	***<0*.*00001***
	**Sensitivity analysis** ^**2**^	4	95/153	FE/MD	0%	2.02	1.24 to 2.80	***<0*.*00001***
	**Sensitivity analysis** ^**3**^	3	225/2549	RE/ MD	56%	2.82	1.39 to 4.26	***0*.*0001***
	**Sensitivity analysis** ^**4**^	4	220/2541	FE/MD	0%	3.29	2.06 to 4.53	***<0*.*00001***

FE: Fixed Effect; RE: Random Effect; MD: Mean Difference; CI: Confidence interval

^1^: Sensitivity analysis excluding data sets with mean or SD values estimated

^2^: Sensitivity analysis excluding the National database study by Tyler et al.

^3^: Sensitivity analysis excluding the studies without at least 20 patients for each treatment arms

^4^: Sensitivity analysis excluding the randomized clinical trial by Park et al.

**Table 5:** Results of the sensitivity analysis for dichotomous outcomes.

Outcomes	Group or Subgroup	Set of data	N patients, RC/LC	Analysis model / Effect measure	I^2^	Summary Statistics	95% CI	P value
**Conversion to open**	**Sensitivity analysis** ^**3**^	10	536/907	FE/OR	20%	0.65	0.36 to 1.15	0.14
	**Sensitivity analysis** ^**3**^ ^**,**^ ^*^	10	536/907	FE/ RD	24%	-0.02	-0.04 to 0.00	0.11
	**Sensitivity analysis** ^**4**^	12	549/946	FE/OR	20%	0.67	0.39 to 1.15	0.15
	**Sensitivity analysis** ^**4**^ ^**,**^ ^*^	12	549/946	FE/RD	27%	-0.02	-0.04 to 0.00	0.12
**Overall postoperative complications**	**Sensitivity analysis** ^**3**^	10	536/907	FE/ OR	1%	0.73	0.56 to 0.95	***0*.*02***
	**Sensitivity analysis** ^**4**^	11	549/947	FE/OR	0%	0.73	0.56 to 0.95	***0*.*02***
**Anastomotic leak**	**Sensitivity analysis** ^**3**^	10	536/907	FE/ OR	0%	0.63	0.32 to 1.25	0.19
	**Sensitivity analysis** ^**3**^ ^**,**^ ^*^	10	536/907	FE/ RD	9%	-0.01	-0.03 to 0.00	0.13
	**Sensitivity analysis** ^**4**^	12	549/946	FE/OR	0%	0.65	0.34 to 1.24	0.19
	**Sensitivity analysis** ^**4**^ ^**,**^ ^*^	12	549/946	FE/RD	0%	-0.01	-0.03 to 0.00	0.14
**Postoperative ileus**	**Sensitivity analysis** ^**2**^	11	449/789	FE/ OR	4%	0.61	0.34 to 1.11	0.11
	**Sensitivity analysis** ^**2**^ ^**,**^ ^*^	11	449/789	FE/ RD	10%	-0.02	-0.05 to -0.00	0.09
	**Sensitivity analysis** ^**3**^	9	561/3138	FE/ OR	8%	0.74	0.50 to 1.11	0.14
	**Sensitivity analysis** ^**3**^ ^**,**^ ^*^	9	561/3138	FE/ RD	19%	-0.02	-0.04 to -0.00	0.12
	**Sensitivity analysis** ^**4**^	11	574/3177	FE/OR	4%	0.71	0.48 to 1.04	0.08
	**Sensitivity analysis** ^**4**^ ^**,**^ ^*^	11	574/3177	FE/RD	10%	-0.02	-0.05to 0.00	0.06
**Wound Infection**	**Sensitivity analysis** ^**3**^	10	536/907	FE/ OR	0%	0.60	0.36 to 1.01	0.06
	**Sensitivity analysis** ^**3**^ ^**,**^ ^*^	10	536/907	FE/ RD	0%	-0.02	-0.05 to -0.00	***0*.*04***
	**Sensitivity analysis** ^**4**^	12	547/939	FE/OR	0%	0.57	0.33 to 0.97	***0*.*04***
	**Sensitivity analysis** ^**4**^ ^**,**^ ^*^	12	547/939	FE/RD	0%	-0.03	-0.05 to -0.00	***0*.*03***

FE: Fixed Effect; RE: Random Effect; OR: Odds Ratio; RD: Risk Difference; CI: Confidence interval

^2^: Sensitivity analysis excluding the National database study by Tyler et al.

^3^: Sensitivity analysis excluding the studies without at least 20 patients for each treatment arms

^4^: Sensitivity analysis excluding the randomized clinical trial by Park et al.

*: Analysis performed with the Risk Difference (RD) as a summary statistic to also include in the estimated effect the studies with dichotomous outcomes with 0 events in each of the treatment groups.

### Meta-analysis results for the main analysis (performed considering all included studies) and for cancer patients subgroup

#### Length of hospital stay

**Fig 2:**
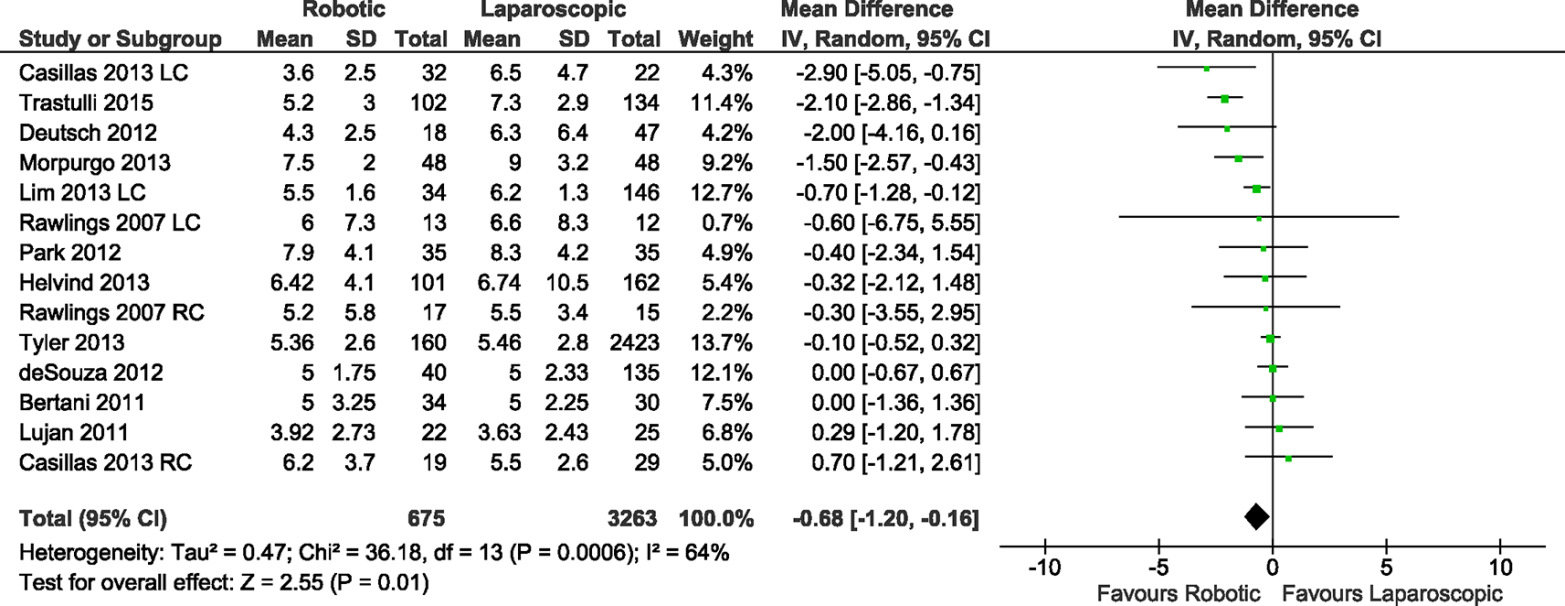
Forest plot of length of hospital stay outcome. RC: Right colectomies data sets; LC: Left colectomies data sets; IV: Inverse Variance; CI: Confidence Interval.

**Fig 3:**
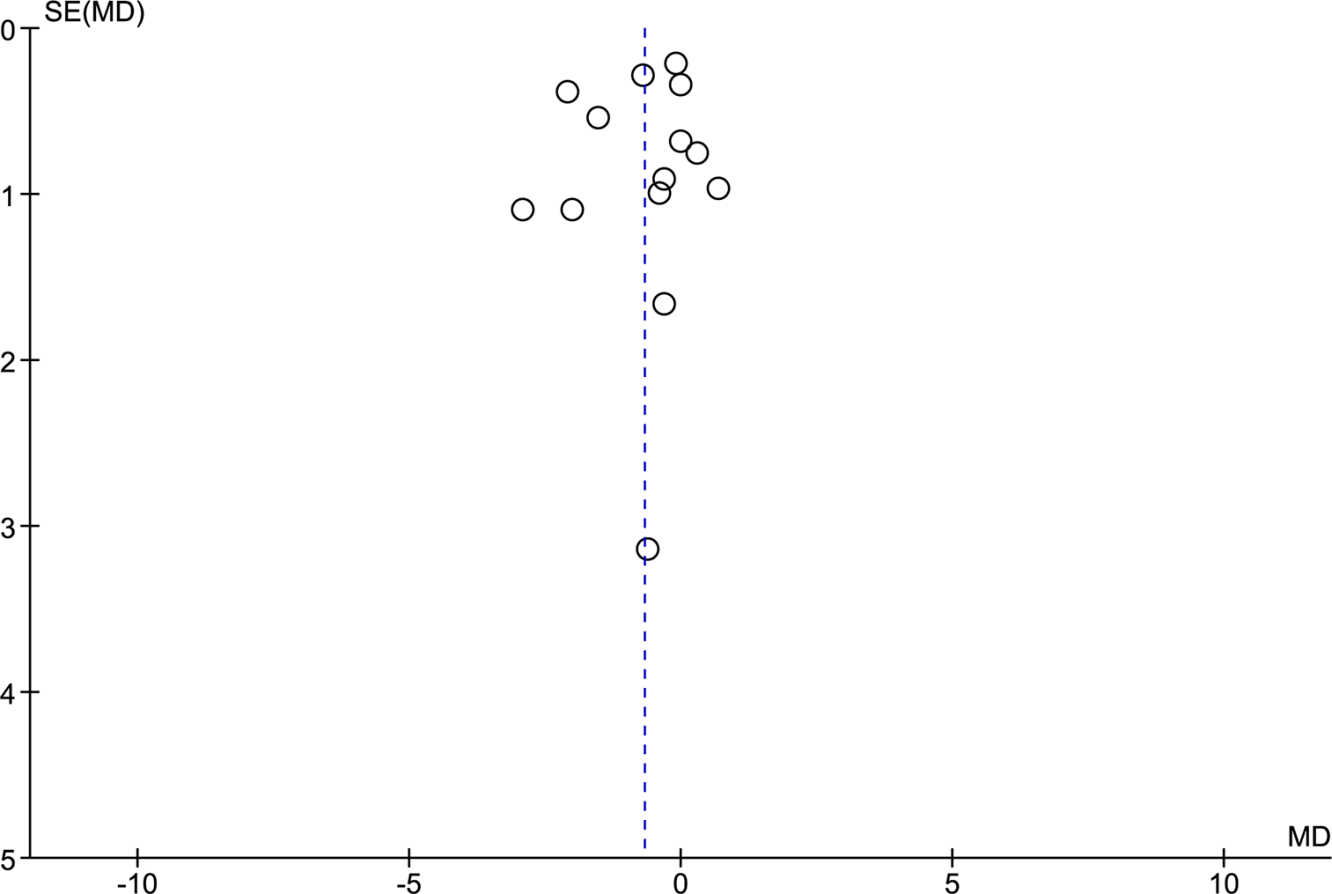
Funnel plot of length of hospital stay outcome. SE: Standard Error; MD: Mean Difference.

All of the included studies reported the length of hospital stay, with a total of 14 data sets, which included 675 patients who underwent RC and 3,263 patients who underwent LC. After pooled analysis (Fig 2), RC resulted in a significantly shorter length of hospital stay than LC (RE, MD -0.68, 95%CI -1.20 to -0.16, P = 0.01); there was evidence of high heterogeneity (X^2^ = 36.18, I^2^ = 64%) but no publication bias (Fig 3). A subgroup analysis that considered only the cancer patients showed a shorter length of hospital stay for patients who underwent RC (FE, MD -0.65, 95%CI -1.09 to -0.22, P = 0.003) and low heterogeneity (X^2^ = 5.45, I^2^ = 8%).

#### Overall postoperative complications

**Fig 4:**
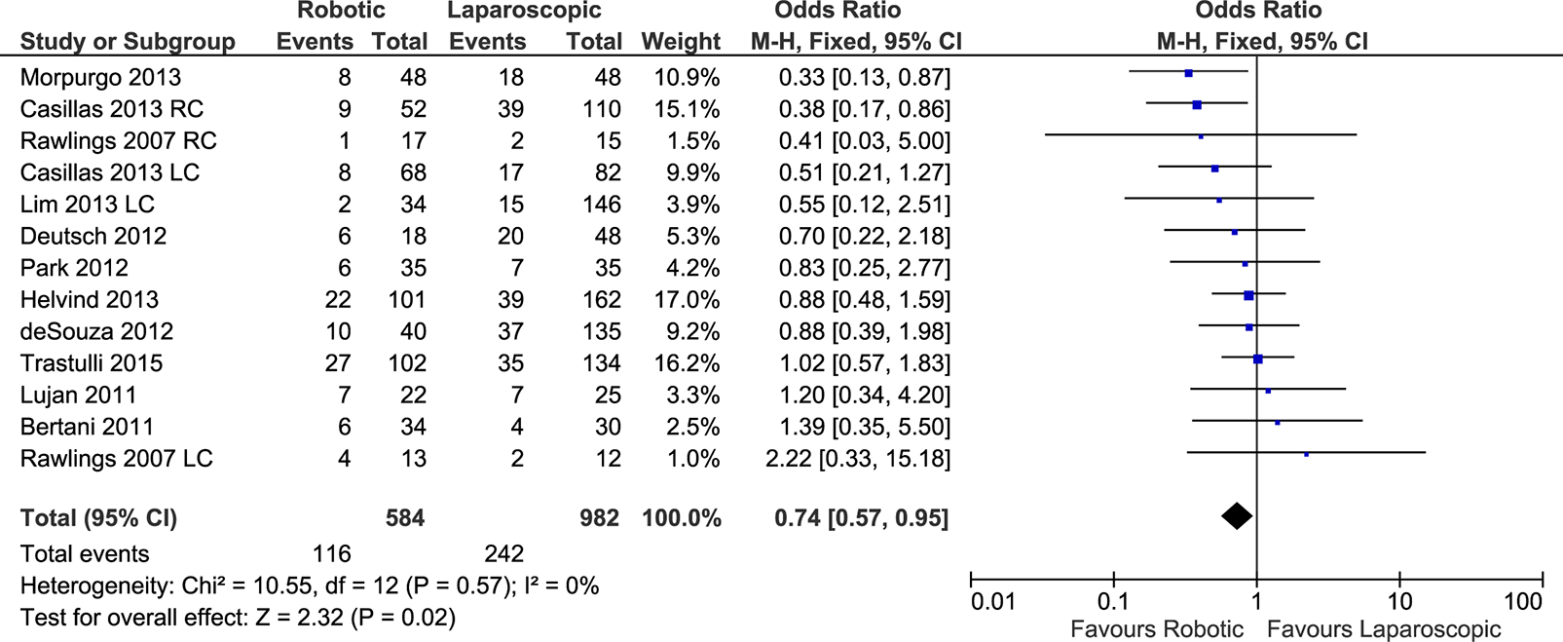
Forest plot of overall postoperative complications outcome. RC: Right colectomies data sets; LC: Left colectomies data sets; M-H: Mantel-Haenszel; CI: Confidence Interval.

**Fig 5:**
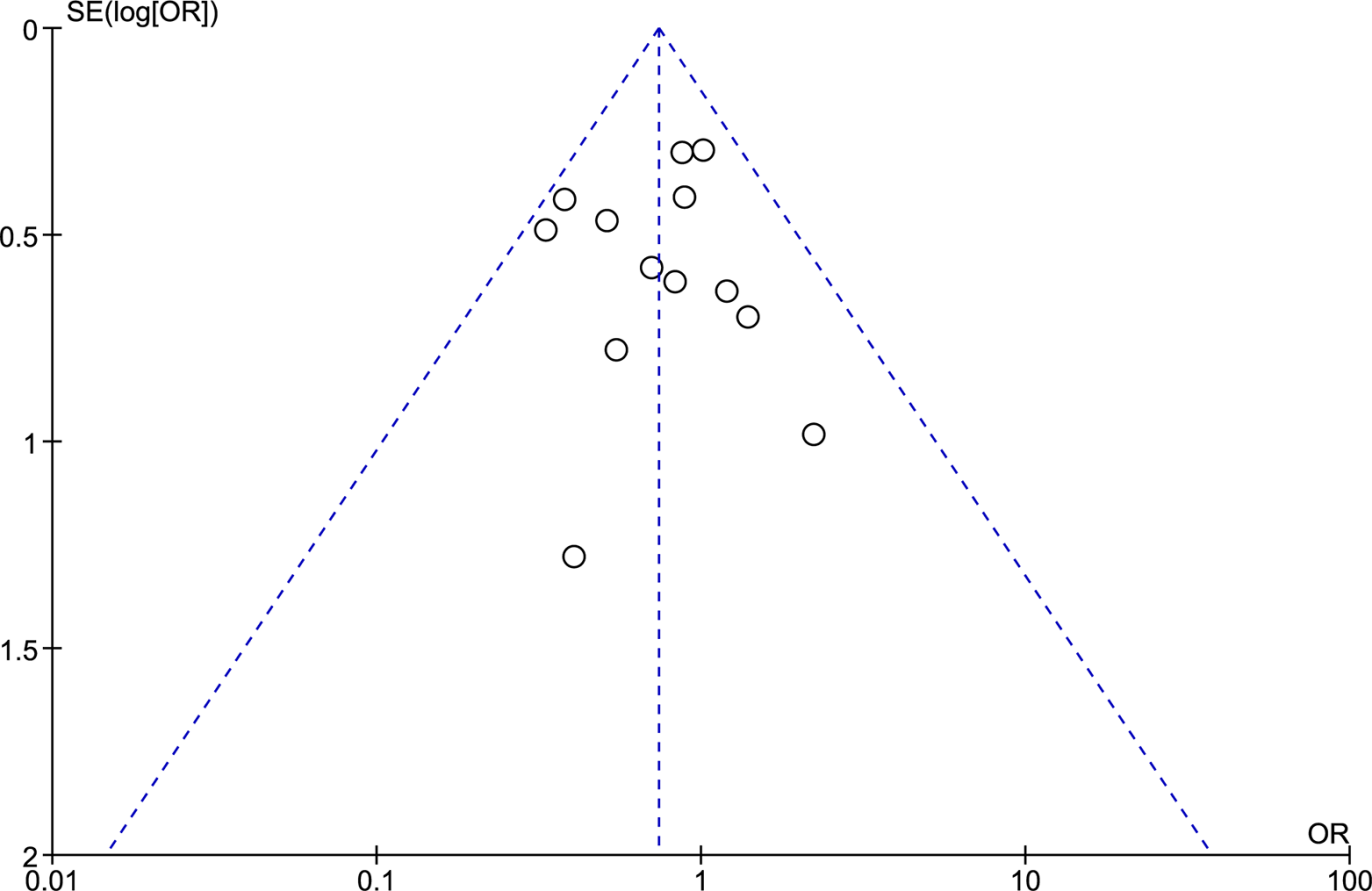
Funnel plot of overall postoperative complications outcome. SE: Standard Error; OR: Odds ratio.

The number of overall postoperative complications was reported in thirteen data sets, from 11 included studies with a total of 584 patients in the RC group and 982 in the LC group. The weighted rate of overall postoperative complications was 21% for the RC group and 26% for the LC group. The meta-analysis (Fig 4) showed significant difference in the postoperative complications between the RC group and the LC group (FE, OR 0.74, 95%CI 0.57 to 0.95, P = 0.02) without heterogeneity (X^2^ = 10.55, I^2^ = 0%) and no evidence of publication bias (Fig 5). In cancer patients, RC resulted in significantly fewer postoperative complications (FE, OR 0.62, 95%CI 0.43 to 0.90, P = 0.01), with moderate heterogeneity (X^2^ = 5.87, I^2^ = 15%).

#### Operative time

**Fig 6:**
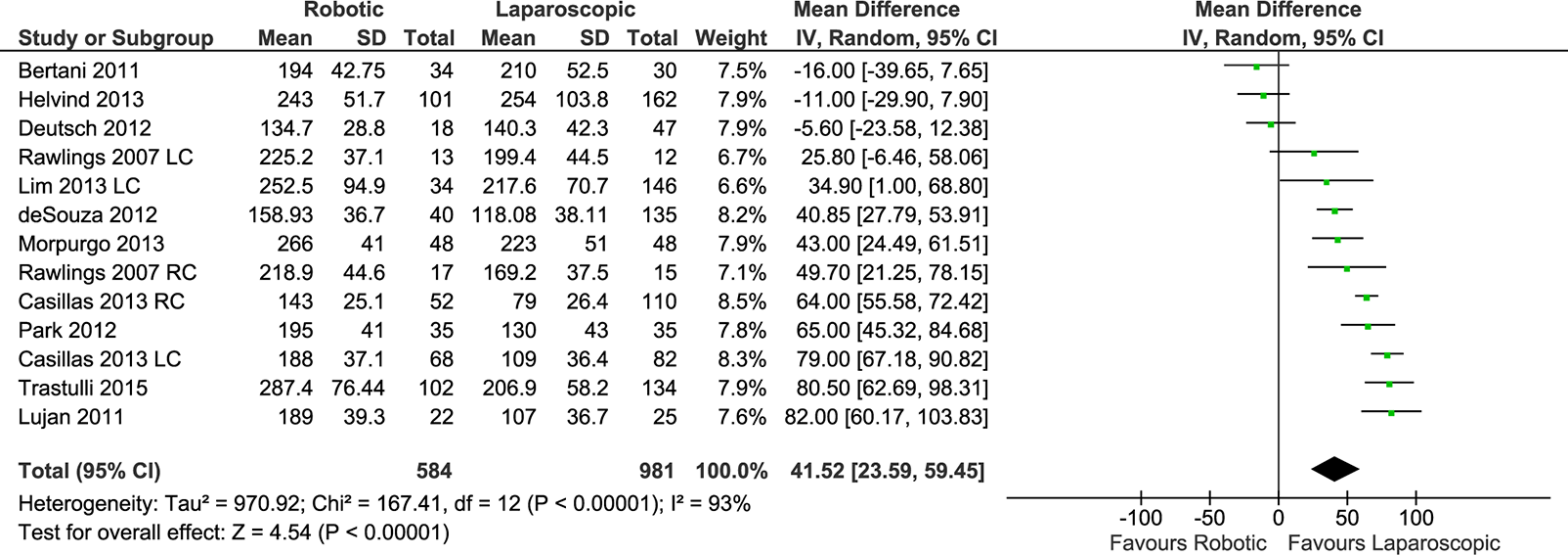
Forest plot of operative time outcome. RC: Right colectomies data sets; LC: Left colectomies data sets; IV: Inverse Variance; CI: Confidence Interval.

In a total of 13 data sets, the operative time was reported for a total of 584 patients in the RC group and 981 in the LC group. The meta-analysis showed a significantly longer operative time for patients who underwent RC (RE, MD 41.52, 95%CI 23.59 to 59.45, P<0.00001), with high heterogeneity (X^2^ = 167.41, I^2^ = 93%) (Fig 6) and no evidence of publication bias.

In the cancer subgroup analysis the operative time was significantly longer for patients who underwent RC (RE, MD 30.47, 95%CI 0.71 to 60.24, P = 0.04) with high heterogeneity (X^2^ = 82.96, I^2^ = 94%).

#### Estimated intraoperative blood loss

**Fig 7:**
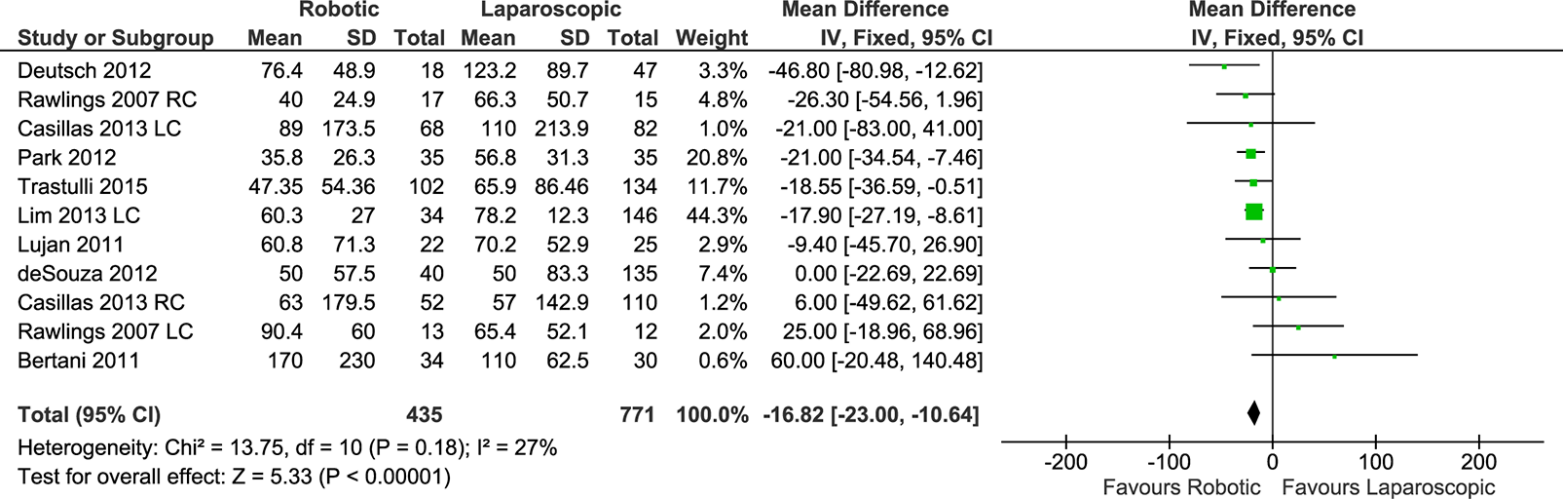
Forest plot of estimated intraoperative blood loss outcome. RC: Right colectomies data sets; LC: Left colectomies data sets; IV: Inverse Variance; CI: Confidence Interval.

Eleven data sets reported on intraoperative blood loss, with a total of 435 patients in the RC group and 771 patients in the LC group. The pooled analysis showed significantly less blood loss in the RC procedures than in the laparoscopic approach (FE, MD -16.82, 95%CI -23.00 to -10.64, P<0.00001), with mild heterogeneity (X^2^ = 13.75, I^2^ = 27%) (Fig 7) and no evidence of publication bias.

The subgroup analysis of cancer patient procedures (4 data sets) showed significantly less blood loss in the RC procedures than in the laparoscopic approach (FE, MD -17.74, 95%CI -25.29 to -10.18, P<00000.1) with moderate heterogeneity (X^2^ = 4.51, I^2^ = 33%).

#### Time to first flatus

**Fig 8:**
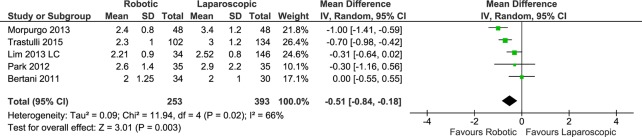
Forest plot of time to first flatus outcome. RC: Right colectomies data sets; LC: Left colectomies data sets; IV: Inverse Variance; CI: Confidence Interval

Five data sets reported the time before the emission of the first flatus (253 patients in the RC group and 393 in the LC group). The meta-analysis showed a significantly shorter time to first flatus in the RC group than in the LC group (RE, MD -0.51, 95%CI -0.84 to -0.18, P = 0.003), with heterogeneity (X^2^ = 11.94, I^2^ = 66%) (Fig 8) but with a symmetrical funnel plot. By contrast, in the subgroup analysis of the four data sets for cancer patients, no significant difference was found between the treatment groups (RE, MD -0.43, 95%CI -0.89 to 0.02, P = 0.06), with higher heterogeneity (X^2^ = 10.32, I^2^ = 71%).

#### Conversion to open surgery

Thirteen data sets from 11 studies reported the rate of robotic and laparoscopic procedures that converted to open surgery (584 patients in the RC group and 981 in the LC group). The weighted rate of conversion to open surgery was 4.3% in the RC group versus 7.1% in the patients who underwent LC.

**Fig 9:**
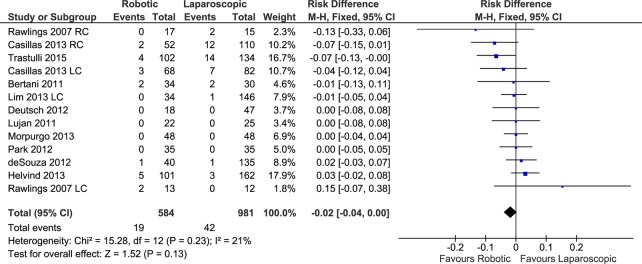
Forest plot of conversion to open surgery outcome. RC: Right colectomies data sets; LC: Left colectomies data sets; M-H: Mantel-Haenszel; CI: Confidence Interval

The pooled analysis showed no significant difference between the compared groups (Fig 9) (FE, RD -0.02, 95%CI -0.04 to 0.00, P = 0.13), with moderate heterogeneity (X^2^ = 15.28, I^2^ = 21%) and no evidence of publication bias.

In the subset of patients with cancer, no differences were found in the rate of conversion to open surgery between the robotic and laparoscopic approaches (FE, RD -0.00, 95%CI -0.03 to 0.02, P = 0.72) with low heterogeneity (X^2^ = 5.03, I^2^ = 1%).

#### Number of harvested lymph nodes

**Fig 10:**
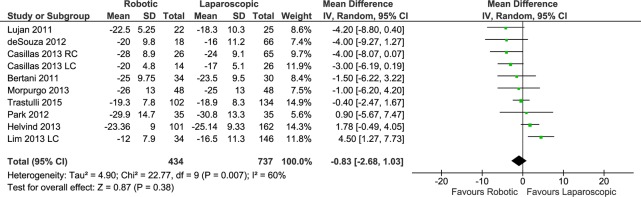
Forest plot of number of harvested lymph nodes outcome. RC: Right colectomies data sets; LC: Left colectomies data sets; IV: Inverse Variance; CI: Confidence Interval. Mean values have been multiplied by -1 in order to graphically invert the direction of the analysis effect.

This outcome was reported in a total of 9 studies, with 10 data sets (434 patients in the RC group versus 737 in the LC group). The number of harvested lymph nodes was similar in the patients undergoing RC and LC (RE, MD -0.83, 95%CI -2.68 to 1.03, P = 0.38), with heterogeneity (X^2^ = 22.77, I^2^ = 60%) (Fig 10) and no evidence of publication bias.

Considering only the procedures for cancer, RC resulted in no significant advantage over LC (RE, MD -0.22, 95%CI -2.27 to 1.83, P = 0.84) with heterogeneity (X^2^ = 16.91, I^2^ = 59%).

#### Anastomotic leak

**Fig 11:**
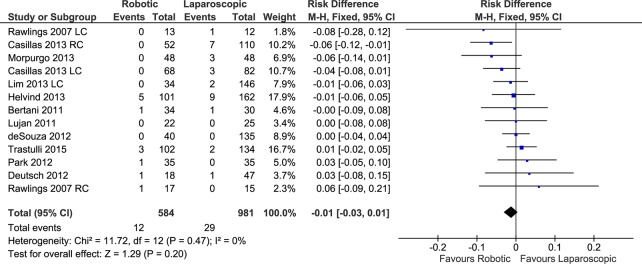
Forest plot of anastomotic leak outcome. RC: Right colectomies data sets; LC: Left colectomies data sets; M-H: Mantel-Haenszel; CI: Confidence Interval.

Eleven studies with a total of 13 data sets reported the number of anastomotic leaks after robotic and laparoscopic colectomies (total of 584 patients in the RC group and 981 in the LC group). The weighted rate of anastomotic leak was 3.2% after RC and 4.1% after LC, although no significant difference was found after a meta-analysis of the data (FE, RD -0.01, 95%CI -0.03 to 0.01, P = 0.20), with low heterogeneity (X^2^ = 11.72, I^2^ = 0%) (Fig 11) and no evidence of publication bias.

Analyzing only the procedures performed for cancer, there was no difference between RC and LC in terms of anastomotic leak (FE, OR 0.58, 95%CI 0.26 to 1.29, P = 0.18) without heterogeneity.

#### Postoperative ileus

**Fig 12:**
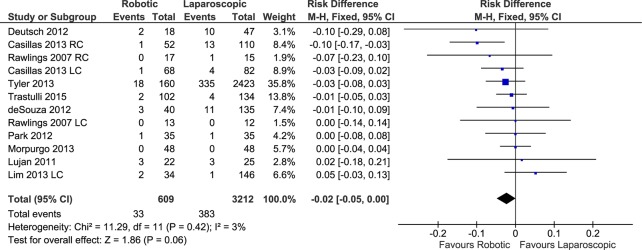
Forest plot of postoperative ileus outcome. RC: Right colectomies data sets; LC: Left colectomies data sets; M-H: Mantel-Haenszel; CI: Confidence Interval.

Ten studies with a total of 12 data sets reported the number of cases of postoperative ileus after robotic and laparoscopic colectomies (a total of 609 patients in the RC group and 3,212 in the LC group). The weighted rate of postoperative ileus was 7.6% after RC and 13% after LC. The pooled analysis showed no significant difference between the compared groups (FE, RD -0.02, 95%CI -0.05 to 0.00, P = 0.06) (Fig 12), with no evidence of heterogeneity (X^2^ = 11.29, I^2^ = 3%) or publication bias.

Considering only the procedures performed for cancer, there was no difference between RC and LC in terms of postoperative ileus (RE, RD -0.01, 95%CI -0.07 to 0.04, P = 0.67) with heterogeneity (X^2^ = 9.05, I^2^ = 67%).

#### Wound Infection

**Fig 13:**
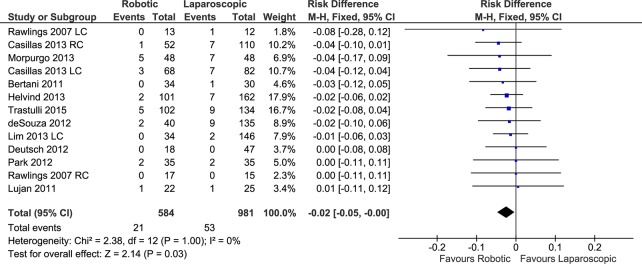
Forest plot of wound infection outcome. RC: Right colectomies data sets; LC: Left colectomies data sets; M-H: Mantel-Haenszel; CI: Confidence Interval

Eleven studies with a total of 13 data sets reported the number of wound infections after robotic and laparoscopic colectomies (total of 584 patients in the RC group and 981 in the LC group). The weighted rate of wound infection was 4.7% after RC and 6.4% after LC. A significant difference was found after pooling the data (FE, RD -0.02, 95%CI -0.05 to -0.00, P = 0.03) (Fig 13), with no evidence of heterogeneity (X^2^ = 2.38, I^2^ = 0%) or publication bias. Considering only the procedures for cancer, there was no difference between RC and LC in terms of wound infection (FE, OR 0.54, 95%CI 0.26 to 1.14, P = 0.11) without heterogeneity.

#### Costs

**Fig 14:**
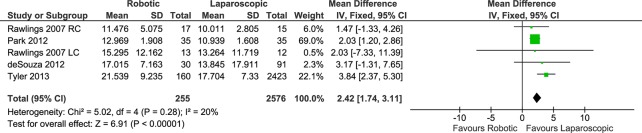
Forest plot of costs outcome. RC: Right colectomies data sets; LC: Left colectomies data sets; IV: Inverse Variance; CI: Confidence Interval. Data in US Dollars.

Data on overall costs were reported in a total of 5 data sets (4 studies) that included a total of 255 patients in the RC group and 2,576 patients in the LC group. A meta-analysis of data showed that RC was significantly more expensive than LC (FE, MD 2.42, 95%CI 1.74 to 3.11, P<0.00001), with no significant heterogeneity (X^2^ = 5.02, I^2^ = 20%) (Fig 14) or evidence of publication bias.

The subgroup analysis of procedures for cancer was reported in only one study (FE, MD 2.03, 95%CI 1.20 to 2.86, P<0.00001).

### Results of methodological quality assessment

After a methodological assessment using the 21-point modified Scottish Intercollegiate Guidelines Network scale, 6 of the included studies [13, 15, 18, 36–38] had a fair quality, with ≥ 8 points (mean 12.7 points), whereas the remaining studies [16, 17, 35, 39, 40] were of good methodological quality, with ≥ 14 points (mean 16.7). No studies resulted in poor methodological quality (< 8 points).

**Fig 15:**
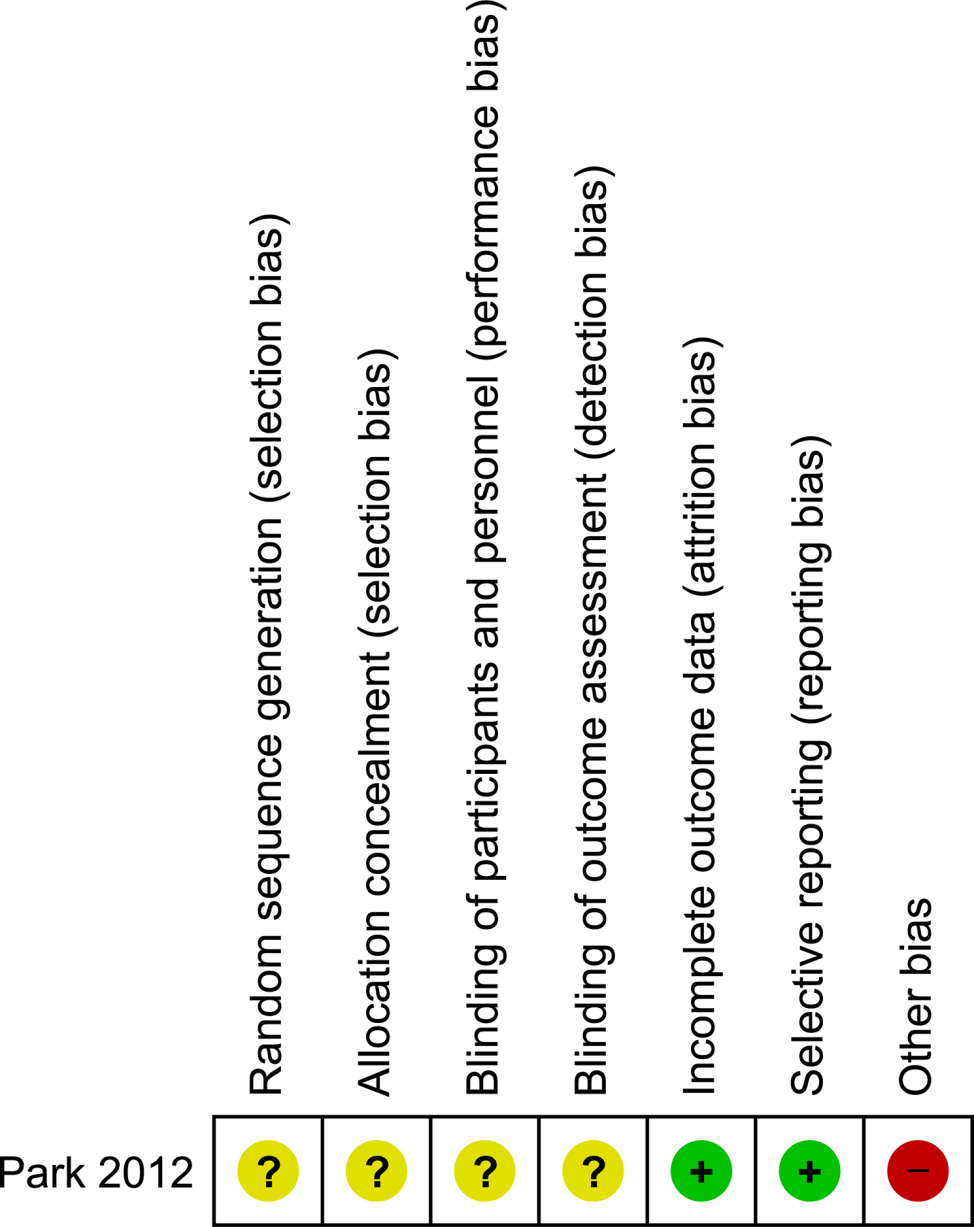
Risk of bias assessment of randomized clinical trials. + Low risk of bias;—High risk of bias;? Unclear risk of bias

The only included randomized clinical trial, by Park et al. [14], had good quality, as determined by assessment with the modified Jadad scale, with a total of 11 points. The results of the assessment of the risk of bias for the RCT by Park et al. are shown in Fig 15.

### Meta-analysis results for the right colectomy and left colectomy subgroups

The results of the subgroup analysis are summarized in Tables 2 and 3, respectively.

#### Length of hospital stay

In patients undergoing robotic right colon resections, the length of hospital stay was shorter than in the laparoscopic group, but the difference was not significant (RE, MD -0.74, 95%CI -1.61 to 0.13, P = 0.10), and heterogeneity was evident (X^2^ = 24.14, I^2^ = 71%). By contrast, the patients undergoing robotic left colectomy had a significant shorter length of stay than the laparoscopic patient group (FE, MD -0.85, 95%CI -1.40 to -0.29, P = 0.003), with evidence of moderate heterogeneity, but the analysis included only three data sets (a total of 259 patients).

#### Overall postoperative complications

Considering only the right colon resection, the robotic approach resulted in significantly fewer postoperative complications than did the laparoscopic approach (FE, OR 0.70, 95%CI 0.50 to 0.96, P = 0.03), with low heterogeneity evident. Considering only the 3 studies reporting data on left colectomies, we found no significant difference in postoperative complications between the robotic and laparoscopic approaches (FE, OR 0.64, 95%CI 0.32 to 1.29, P = 0.21) without heterogeneity (X^2^ = 1.89, I^2^ = 0%).

#### Operative time

In patients who underwent robotic right colon resections, the operative time was significantly longer (RE, MD 52.32, 95%CI 34.21 to 70.43, P<0.00001), with slightly less heterogeneity evident (X^2^ = 68.97, I^2^ = 90%).

This outcome was compared for left colectomy in only 3 data sets. A pooling of the data showed a significantly shorter operative time for LC (RE, MD 49.01, 95%CI 10.53 to 87.49, P = 0.01), with heterogeneity (X^2^ = 13.52, I^2^ = 85%).

#### Estimated intraoperative blood loss

The subgroup analysis for right colectomies showed significantly less blood loss for the robotic approach (FE, MD -18.28, 95%CI -26.84 to -9.73, P<0.0001), with low heterogeneity (X^2^ = 6.60, I^2^ = 9%). Only 3 data sets reported this outcome for left colectomy (FE, MD -16.17, 95%CI -25.16 to -7.17, P = 0.0004), and these showed significantly less blood loss for the robotic approach with moderate heterogeneity (X^2^ = 3.53, I^2^ = 43%).

#### Time to first flatus

The time to first flatus was significantly shorter for the RC patients in the subgroup analysis of right colon procedures (FE, MD -0.76, 95%CI -0.99 to -0.54, P<0.00001), with less heterogeneity (X^2^ = 2.59, I^2^ = 23%). A total of one study reported this outcome for left colectomy, and it showed no differences between the two groups (FE, MD -0.31, 95%CI -0.64 to 0.02, P = 0.06).

#### Conversion to open surgery

Considering the subgroup of right colon procedures, we found significant differences between the investigated procedures (FE, RD -0.03, 95%CI -0.06 to -0.01, P = 0.02), with moderate heterogeneity (X^2^ = 12.64, I^2^ = 45%). This outcome was compared for left colectomy in a total of 3 data sets. A pooling of the data showed no difference between the robotic and laparoscopic procedures (FE, OR 0.87, 95%CI 0.29 to 2.57, P = 0.80), without heterogeneity.

#### Number of harvested lymph-nodes

In the robotic right colon resections, the number of harvested lymph nodes was significantly higher than in the laparoscopic approach (FE, MD -1.58, 95%CI -3.09 to -0.07, P = 0.04), with no heterogeneity (X^2^ = 5.26, I^2^ = 5%).

Only two studies reported this outcome for left colectomy, and they showed no differences between the two groups (RE, MD 0.75, 95%CI -6.60 to 8.10, P = 0.84) with high heterogeneity.

#### Anastomotic leak

After right colectomy, no differences were found in anastomotic leak rate between the robotic and laparoscopic procedures (FE, RD -0.01, 95%CI -0.03 to 0.02, P = 0.51), with moderate heterogeneity.

The number of anastomotic leaks was reported in only 3 studies on left colectomy, and after pooling the data, no difference was found between the robotic and laparoscopic procedures (FE, OR 0.31, 95%CI 0.05 to 1.84, P = 0.20), without heterogeneity.

#### Postoperative ileus

After the subgroup analysis for right colectomy, no differences were found in postoperative ileus between the robotic and laparoscopic procedures (FE, RD -0.03, 95%CI -0.06 to 0.00, P = 0.05), with low heterogeneity.

The postoperative ileus incidence was reported in only 3 studies on left colectomy, and after pooling the data, no difference was found between the robotic and laparoscopic procedures (FE, RD 0.00, 95%CI -0.04 to 0.05, P = 0.92), with moderate heterogeneity.

#### Wound Infection

After right colectomy, no differences in wound infection were found between the robotic and laparoscopic procedures (FE, RD -0.02, 95%CI -0.05 to 0.01, P = 0.21), without heterogeneity.

Wound infections were reported in only 3 studies on left colectomy, and after pooling the data, no significant difference was found between the robotic and laparoscopic procedures (FE, OR 0.50, 95%CI 0.15 to 1.62, P = 0.25), without heterogeneity.

#### Costs

The subgroup analysis for right colectomy showed that RC was significantly more expensive than LC (FE, MD 2.02, 95%CI 1.24 to 2.80, P<0.00001), with less heterogeneity (X^2^ = 0.40, I^2^ = 0%). The data on the cost of left colectomy were available from only 1 data set and indicated no significant difference between the RC and LC approaches (FE, MD 2.03, 95%CI -7.33 to 11.39, P = 0.67).

## Results of the Sensitivity Analysis

The results of the planned sensitivity analysis performed for each of the considered primary and secondary outcomes, summarized in Tables 4 and 5, were all consistent with the results of the main analysis confirming the stability of our results. Due to the low number of patients undergoing single port access in the study by Deutsch et al. [38] we considered not useful to perform the sensitivity analysis by excluding this study from the main analysis.

## Discussion

Our systematic review and meta-analysis suggests that compared with a laparoscopic approach, a robotic colectomy provides a significantly shorter time to first flatus, a shorter length of hospital stay, less intraoperative blood loss and a significant reduction in the rate of overall postoperative complications and wound infections.

We found no statistically significant differences in the conversion to open surgery rate, number of harvested lymph nodes, rate of both anastomotic leak and postoperative ileus, but we did find that RC required a higher operative time and cost than did laparoscopy.

The results of the meta-analysis for the subgroup of patients with cancer showed a significant advantage of RC in the overall postoperative complications rate, in the intraoperative estimated blood loss and in the length of hospital stay. Laparoscopic approach demonstrated shorter operative time and lower costs. No differences were found between the two approaches in the analyses of the remaining outcomes.

Considering only the subgroup analysis for the right colectomy procedures, RC resulted in significantly less intraoperative blood loss, a shorter time to first flatus, a lower conversion to open rate, a lower overall postoperative complication rate, and a significantly higher number of harvested lymph nodes. Right RC required longer operative times and higher costs than the laparoscopic approach.

Although we also performed a subgroup analysis considering only the left colectomy procedures, the number of studies and data sets that reported data for this procedure was small (only 3 studies for a total of 355 patients), thereby precluding any meaningful conclusion.

Based on our analysis, the robotic approach to colonic surgery provides a faster recovery of bowel function and a hospital stay that was approximately 1 day shorter than that required for LC. These RC advantages are not difficult to accept if we consider some of the robotic technical characteristics. We hypothesize that the shorter length of stay in the RC group could be explained by the improved ergonomics, the avoidance of the “fulcrum effect” and the more precise instrumental maneuvers in the robotic technique, which are less traumatic to the viscera and tissues and allow minor stretching of the mesentery during the dissection steps. RC also allows for fine tissue dissection, in contrast to laparoscopy. This difference could likely translate to a faster recovery of bowel function, which consequently could contribute to a faster discharge.

Our study demonstrates evidence for a faster recovery of bowel function after robotic colectomy, including time to first flatus emission and discharge 1 day earlier than laparoscopy which were significant.

However, it is obvious that the length of hospital stay and other soft endpoints, such as time to first flatus, are notably prone to risk of performance and selection bias, in particular in observational studies.

In regard to postoperative complications, we found a significantly lower overall complication rate for robotic colectomy in the main analysis, in the subgroup analysis of patients undergoing right colon resections and in those who underwent colonic resection for cancer. In particular the robotic approach provided a significant reduction in wound infection rates. Postoperative ileus rate resulted lower in the RC group if compared to laparoscopic approach (weighted rates of 7.6% versus 13% respectively) although this did not quite reach statistical significance. It is well known that postoperative ileus is one of the most common causes of delayed discharge for patients after colorectal surgery [43]. The likely reduction in the postoperative ileus rate after RC would, if confirmed in future studies, be an interesting finding, particularly because postoperative ileus is considered to be a relevant predictor of hospital resource utilization after colon surgery [43–45].

In general, the reduction in the incidence of postoperative complications in the subgroup of patients undergoing robotic right colonic resection and resection for cancer could potentially be explained by the advantages related to the use of the robotic platform, which are hypothetically more evident in right hemicolectomy, which requires a wide range of colon resection and involves a more complex vascular anatomy than left colectomy. Patients with cancer, in whom it is necessary to follow the principles of oncological radicality (complete mesocolic excision), required a more aggressive surgical approach. The magnification of the operative field and the precise instrument control (allowing a more fine and gentle tissue manipulation and dissection) could also explain the significantly lower intraoperative blood loss in the robotic procedures and in the subgroup analysis for right colectomies, left colectomies and colectomies for cancer.

Some authors have suggested [10, 16] that the fine and meticulous dissection provided by the magnified field of vision and increased dexterity allowed by the robot facilitates the performance of a lymphadenectomy.

However, a significant advantage in the number of retrieved lymph nodes in the patients who received robotic surgery was found only in the subgroup of patients undergoing robotic right colectomies.

Our analysis showed a significant reduction in wound infections after the robotic colectomies compared with laparoscopic ones. This finding is not easy to explain because surgical site infections are influenced by many factors. It is well known that laparoscopic colorectal surgery results in less immunosuppression than does open surgery [46–48]. Reducing surgical stress by attenuating the neuro-hormonal response to surgical trauma represents a factor for enhanced recovery and reduces the risk of complications and infections [49, 50]. As previously stated, robotic surgery could produce even less surgical trauma than laparoscopy and could allow for more attenuated surgery-induced immunosuppression, which would have a positive impact on the predisposition of the patient to surgical wound infections.

Our meta-analysis found a significant reduction in the conversion to open surgery rate in favor of the robotic approach only in the subgroup analysis of right colectomy. However, owing to the large number of observational studies included in this meta-analysis, we hypothesize that this finding could be related, in part, to the possibility that the patients selected by the surgeons for the robotic approach could be “highly selected patients” compared with those undergoing laparoscopic colectomies.

As expected, our analysis revealed a significantly longer operative time and higher cost for the robotic approach than for laparoscopy, and these results were confirmed in the subgroup analysis for right colectomies, left colectomies and cancer resections.

To date, only the Da Vinci Robot system is approved by the US Food and Drug Administration and has notably higher acquisition and maintenance costs than laparoscopic equipment. The future entry of new surgical robotic devices into the marketplace could drastically reduce the prices and aid technological progress [51].

It is fundamental to highlight that this meta-analysis has some limitations. These limits include, in particular, the observational and retrospective design of the larger portion of the included studies that exposes the analysis to a risk of bias. However, all of the included studies are of good or acceptable methodological quality based on our assessment, and the performed sensitivity analysis showed the robustness and consistency of the results. We decided to include the national database study by Tyler et al. in our analysis, although these studies may have some peculiar methodological limitations [52]. Its inclusion was to improve the comprehensiveness of the analyzed data, avoid the loss of significant information, and increase both the sample size and the power of the meta-analysis. To date, there is no consensus about the inclusion or exclusion of these types of observational studies and some authors have included these studies in their meta-analyses [53–55].

For these reasons a sensitivity analysis excluding the study by Tyler et al. was performed in the present meta-analysis. It demonstrated that the inclusion of the data from this study did not modify the results of the analysis in terms of the estimated effect size, the heterogeneity, or the statistical significance of the outcomes for which data from this study were available (only postoperative length of stay, cost and postoperative ileus outcomes).

Performing a meta-analysis of data retrieved from studies with different designs is another limitation, but overall, including studies with different designs allowed us to increase the statistical power and the external validity of the findings.

Considering these limits, the results of this meta-analysis, although new and overall favoring the robotic approach, should be considered with caution and are not sufficient to justify the routine use of robotic technology for elective colon surgery.

Finally, this meta-analysis, which was primarily based on observational studies, can be considered a useful tool “to understand and quantify sources of variability in results across studies” [56] and to help in the planning of future RCTs, which are essential to definitively identify the role of the robotic approach in the field of colonic surgery.

## Supporting Information

S1 Table Technical characteristics of the included studies. (DOCX)

S1 Fig PRISMA 2009 Checklist. (DOCX)
