# Supplementary material for: Robotic versus Laparoscopic Approach in Colonic Resections for Cancer and Benign Diseases: Systematic Review and Meta-Analysis
Source: PLoS One. 2015 Jul 27;10(7):e0134062. doi: 10.1371/journal.pone.0134062 (PMC4516360; doi:10.1371/journal.pone.0134062)
Supplement: S1 Table — (DOCX) [file pone.0134062.s001.docx]

**S1 Table: Technical characteristics of the included studies**

| **Author** | **Surgery** | **Approach to dissection** | **Mobilization** | **Vessel Ligation** | **Transection** | **Anastomosis** |
| --- | --- | --- | --- | --- | --- | --- |
| Rawlings^a^ | RC | Medial to lateral | Intracorporeal | Intracorporeal | Intracorporeal | Isoperistaltic side-to-side intracorporeal  Stapled with a handsewn enterotomies closure |
|  | LC | Medial to lateral | Intracorporeal | Intracorporeal | Extracorporeal | Extracorporeal |
| Rawlings^b^ | RC | Medial to Lateral | Intracorporeal | Intracorporeal | Extracorporeal (rectum intra) | End to-end anastomosis with circular stapler |
|  | LC | Medial to Lateral | Intracorporeal | Intracorporeal | Extracorporeal (rectum intra) | End to-end anastomosis with circular stapler |
| de Souza | RC | Either a lateral to medial or a medial to lateral approach | Intracorporeal | Intracorporeally or  extracorporeally* | Extracorporeal | Extracorporeal side-to-side ileocolic  anastomosis with linear stapler |
|  | LC | “The sequence of surgical steps for both the robotic and laparoscopic procedures is identical” | | | | |
| Lujan | RC | Either a lateral to medial or a medial to lateral approach | Intracorporeal | Intracorporeal | Intracorporeal | Intracorporeal stapled with handsewn colosure of the enterotomies or extracorporeal (4 patients) |
|  | LC | Lateral to medial | Intracorporeal | Intracorporeal | Extracorporeal | Side-to-side totally stapled extracorporeal anastomosis |
| Morpurgo | RC | Medial to lateral | Intracorporeal | Intracorporeal | Intracorporeal (ileum and transverse colon) | Intracorporeal stapled with handsewn eterotomies closure |
|  | LC | Medial to lateral | Intracorporeal | Intracorporeal | Extracorporeal | Handsewn latero-lateral extracorporeal anastomosis |
| Casillas^a^ | RC | Medial to lateral | Intracorporeal | Intracorporeal | Extracorporeal | Extracorporeal |
|  | LC | Medial to lateral | Intracorporeal | Intracorporeal | Extracorporeal | Extracorporeal |
| Casillas^b^ | RC | Medial to lateral | Intracorporeal | Intracorporeal | Extracorporeal | End to end with circular stapler |
|  | LC | Medial to Lateral | Intracorporeal | Intracorporeal | Extracorporeal | End to end with circular stapler |
| Deutsch | RC | Medial to lateral | Intracorporeal | Intracorporeal | Extracorporeal | Extracorporeal side to side functional end to end anastomosis |
|  | LC | NR | NR | NR | NR | NR |
| Park | RC | Inferior-to-superior technique | Intracorporeal | Intracorporeal | Intracorporeal | Extracorporeal or intracorporeal |
|  | LC | Inferior-to-superior technique | Intracorporeal | Intracorporeal | Intracorporeal | Extracorporeal or intracorporeal |
| Helvind | RC | Medial and ‘‘vessel first’’ approach | Intracorporeal | Intracorporeal | Extracorporeal | Extracorporeal for right colectomy  Intracorporeal stapled anastomosis  with a circular stapler for left colectomy |
|  | LC | Medial and ‘‘vessel  first’’ approach | Intracorporeal | Intracorporeal | Extracorporeal | Extracorporeal for right colectomy  Intracorporeal stapled anastomosis  with a circular stapler for left colectomy |
| Tyaler | RC | NR | NR | NR | NR | NR |
|  | LC | NR | NR | NR | NR | NR |
| Bertani | RC | Medial to Lateral | Intracorporeal | Intracorporeal | Intracorporeal | Extracorporeal in right colectomy  Intracorporeal transanal double-stapled anastomosis in left colectomies |
|  | LC | Medial to Lateral | Intracorporeal | Intracorporeal | Intracorporeal | Intracorporeal in right colectomies  Intracorporeal transanal double-stapled anastomosis in left colectomies |
| Trastulli | RC | Medial to Lateral | Intracorporeal | Intracorporeal | Intracorporeal | Intracorporeal latero-lateral stapled or handsewn |
|  | LC | Medial to Lateral | Intracorporeal | Intracorporeal | Intracorporeal | Intra or extracorporeal handsewn or stapled anastomosis |

RC: Robotic colectomy; LC: Laparoscopic colectomy; NR: Not Reported; ^a^ Right colectomy data sets; ^b^ Left colectomy data sets; * Intracorporeally or extracorporeally in the lateral to medial approach. When dissecting medial to lateral, the ileocolic and right colic pedicles were divided intracorporeally.
